# The Azurin-Derived Peptide CT-p19LC Exhibits Membrane-Active Properties and Induces Cancer Cell Death

**DOI:** 10.3390/biomedicines9091194

**Published:** 2021-09-10

**Authors:** Ana Rita Garizo, Lígia F. Coelho, Sandra Pinto, Tiago P. Dias, Fábio Fernandes, Nuno Bernardes, Arsénio M. Fialho

**Affiliations:** 1 iBB-Institute for Bioengineering and Biosciences, Biological Sciences Research Group, Instituto Superior Técnico, University of Lisbon, Av. Rovisco Pais, 1, 1049-001 Lisbon, Portugal; anagarizo@tecnico.ulisboa.pt (A.R.G.); ligiapfcoelho@gmail.com (L.F.C.); sandrapinto@ist.utl.pt (S.P.); tiagompdias@tecnico.ulisboa.pt (T.P.D.); fernandesf@tecnico.ulisboa.pt (F.F.); nuno.bernardes@tecnico.ulisboa.pt (N.B.); 2 Associate Laboratory i4HB—Institute for Health and Bioeconomy, Instituto Superior Técnico, University of Lisbon, Av. Rovisco Pais, 1, 1049-001 Lisbon, Portugal; 3 Department of Bioengineering, Instituto Superior Técnico, University of Lisbon, Av. Rovisco Pais, 1, 1049-001 Lisbon, Portugal

**Keywords:** anticancer peptide, azurin, peptide-based drug development, cytotoxic effect, membrane-based anticancer therapy

## Abstract

Peptides have been thoroughly studied as new therapeutic strategies for cancer treatment. In this work, we explored in vitro the anticancer potential of three novel peptides derived from the C-terminal of azurin, an anticancer bacterial protein produced by *Pseudomonas aeruginosa*. CT-p26, CT-p19 and CT-p19LC peptides were previously obtained through an in silico peptide design optimization process, CT-p19LC being the most promising as it presented higher hydrophobicity and solubility, positive total charge and, most importantly, greater propensity for anticancer activity. Therefore, in this study, through proliferation and apoptosis assays, CT-p19LC was tested in four cancer cell lines—A549, MCF-7, HeLa and HT-29—and in two non-cancer cell lines—16HBE14o- and MCF10A. Its membrane-targeting activity was further evaluated with zeta potential measurements and membrane order was assessed with the Laurdan probe. The results obtained demonstrated that CT-p19LC decreases cell viability through induction of cell death and binds to the plasma membrane of cancer cells, but not to non-cancer cells, making them less rigid. Overall, this study reveals that CT-p19LC is an auspicious selective anticancer peptide able to react with cancer cell membranes and cause effective action.

## 1. Introduction

The use of membranolytic anticancer peptides (ACPs) has become a potential strategy for the development of new cancer therapies [1]. ACPs (<10 kDa), either from eukaryotes or of bacterial origin, are small linear or cyclic molecules (5–50 amino acids), rich in cationic and hydrophobic amino acids that give them an overall positive charge (at pH 7) and an amphipathic behavior. These peptides can adopt α-helix or β-pleated sheet configurations, but random coil structures have also been described in the literature [2].

There are two different classifications for ACPs considering their selectivity properties. The first is the ACP_T_ class, which includes non-selective peptides with identical activities against several cell types, such as mammalian, bacterial and cancer cells [3,4]. The second category, named ACP_AO_, corresponds to those that selectively target bacterial and cancer cells while showing residual activity against normal cells. The reason for this behavior is not fully clear yet but the differences at the membrane level between normal and cancer cells may explain, at least in part, this selectivity. In fact, the plasma membrane of cancer cells is characterized as having some unique features, from which a larger surface area, a net negative charge and an abnormal fluidity stand out. This may be due to a high number of microvilli, with the negative charge in the outer layer resulting from the abnormal presence of anionic phospholipid phosphatidylserine, O-glycosylated mucins, sialylated gangliosides and heparin sulfate [3,4,5,6].

The mechanism of action for ACPs leads to the irreparable disruption of the plasma membrane of tumor cells [7] through pore formation, followed by cell lysis (direct-acting mechanism) [8,9]. Both the structure they adopt when in contact with the plasma membrane of these cells as well as their intrinsic characteristics mean that these peptides are capable of associating with this cellular barrier mainly through electrostatic interactions [4,10]. Apart from the plasma membrane, other internal membranes may be targeted by the membranolytic effects of ACPs, such as the mitochondrial membrane, where their effects can trigger apoptosis (indirect-acting mechanism) [8,11].

The development of cancer therapies with the use of ACPs presents advantages for clinical applications compared to conventional chemotherapy. In particular, ACPs act both in metabolically active tumor cells and in slow-growing or multidrug-resistant cancer cells [12]. Additionally, ACPs have a relatively high tissue penetration, the cost for producing them is low and they can be easily modified by solid-phase synthesis technology [13].

Currently, the database of the National Library of Medicine (NLM) at the National Institutes of Health (NIH) in the PubMed.gov platform displays a total of 463 clinical trials with the application of ACPs in several types of cancer, being the most common studies in melanomas, breast and lung cancer [9,14]. As examples, LTX-3158, a human lactoferrin-derived oncolytic peptide, is currently in a phase I clinical trial and bryostatin 1, a peptide within the bryostatin family composed of marine natural products, is at phase II [15,16]. In addition to them, there is p28, a cell-penetrating peptide derived from the anticancer protein azurin (14 kDa) produced by the bacterium *Pseudomonas aeruginosa* [17]. This peptide has already completed two phase I clinical trials in cancer patients [18,19] and received approval as an orphan drug by the Food and Drug Administration (FDA) [20]. Overall, these studies show promising results for the treatment of cancer with ACPs not only as sole drugs but also in combination with other therapeutic approaches [21].

The aim of this study was to evaluate the anticancer potential of new peptides derived from azurin. Evidence from our previous work and from others suggests that azurin may therapeutically act on cancer cell membranes through a lipid raft/*caveolae*-mediated pathway [22,23,24]. By specifically targeting such plasma membrane microdomain sites, azurin promotes a multivalent action accelerating the endocytosis of receptors and the disruption of signaling pathways hyperactivated in cancer cells [25,26]. In addition, it is known that p28, derived from this protein, is a protein transduction domain (PTD), in part responsible for mediating the entrance of azurin into cells, and it also has anticancer properties [25,27]. Beyond this, it has become clear that the anticancer activity exerted by azurin depends on other domains (azurin C-terminal 88–128 amino acids) besides the p28 domain (azurin 50–77 amino acids). In fact, the C-terminal peptide has anticancer activity through its binding with the cell surface EphB2 receptor and interfering in cancer growth promotion, which has been explored to design peptides to improve radiotherapy efficacy in lung cancer [28,29]. On the other hand, the phenylalanine residue at position 114 was found to be critical for azurin uptake by cancer cells [30]. Based on this, in a previous study, our group used a region of 26 amino acid residues of azurin close to its C-terminal (CT-p26 peptide) as a template for the discovery of new bioactive peptides against cancer cells. Bioinformatics tools used in peptide design studies have enabled the assessment of the bioactivity of this native peptide. First, by reducing its length, and then by changing some residues in its amino acid sequence, it was possible to improve the parameters for solubility, hydrophobicity, overall charge and anticancer potential, giving rise to two new peptides, CT-p19 (shorter than CT-p26) and CT-p19LC (three amino acid residues altered compared to CT-p19) [31]. In the present work, we evaluated in vitro the anticancer activity of these peptides and compared it with the anticancer activity of full-length azurin and its derived native peptides.

## 2. Materials and Methods

### 2.1. Azurin-Derived Peptides

The four azurin-derived peptides used, namely p28, CT-p26, CT-p19 and CT-p19LC, were chemically synthetized by Pepmic Co., Ltd., Suzhou, China, with a minimal purity of 95.0%. CT-p19 and CT-p19LC peptides labeled with 5,6-FAM were commercially synthesized by CASLO ApS, Kongens Lyngby, Denmark. Lyophilized samples of the peptides were resuspended in 10 mM sodium phosphate buffer (pH 7.4) or in phosphate buffer saline (PBS; pH 7.4), divided into aliquots and stored at −20 °C.

### 2.2. Circular Dichroism Spectroscopy

The secondary structure of the CT-p19LC peptide was analyzed through spectroscopic analysis. UV-visible and far-UV circular dichroism (CD) spectra were traced. UV-visible spectra between 250 and 800 nm were obtained using a PharmaSpec UV-1700 (Shimadzu, Kyoto, Japan) UV-visible spectrophotometer. Far-UV CD spectra were traced using a Π*-180 spectropolarimeter from Applied Photophysics using default parameters. Ten measurements were made with an integration time of 1 sec, a cuvette path length of 10 mm, a wavelength ranged of 190 to 250 nm and a step size of 1 nm. The obtained spectra were analyzed using the online DICHROWEB server (http://dichroweb.cryst.bbk.ac.uk/html/home.shtml, accessed on 1 July 2019) to predict the secondary structure of the peptide [32].

### 2.3. Human Cancer Cell Lines and Cell Culture Conditions

The A549 (lung), MCF-7 (breast), HeLa (cervix) and HT-29 (colorectal) human cancer cell lines (European Collection of Authenticated Cell Cultures (ECACC), Public Health England, Salisbury, United Kingdom), the 16HBE14o- human bronchial cell line [33] and the MCF10A human mammary gland cell line (American Type Culture Collection (ATCC), Manassas, VA, United States) were used. The cancer cells were seeded and maintained in Dulbecco’s Modified Eagle Medium (DMEM; Gibco^®^ by Life Technologies, Carlsbad, CA, United States). The medium was supplemented with 10% heat-inactivated fetal bovine serum (FBS; Gibco^®^ by Life Technologies, Carlsbad, CA, United States), 100 IU/mL penicillin and 100 mg/mL streptomycin (Pen-Strep, Invitrogen, Waltham, MA, United States). The 16HBE14o- cells were grown in MEM medium without earls’ salts and supplemented with 10% FBS, 1% L-glutamine and 10,000 U/mL penicillin and 10,000 mcg/mL streptomycin (PenStrep, Invitrogen, Waltham, MA, United States). The MCF10A cells were cultured in 50% DMEM/50% F12 nutrient mix, supplemented with 5% equine serum, EGF (20 ng/mL), insulin (10 µg/mL), hydrocortisone (0.5 µg/mL), cholera toxin (100 ng/mL) and 10,000 U/mL penicillin and 10,000 mcg/mL streptomycin (PenStrep, Invitrogen, Waltham, MA, United States). The culture conditions for all cell lines were 37 °C in a humidified chamber containing 5% CO_2_ (binder CO_2_ incubator C150, Keison products, Chelmsford, United Kingdom).

### 2.4. MTT Cell Proliferation Assays

Cell proliferation after treatment with the peptides was measured by MTT (3-(4,5 dimethylthiazol-2-yl-2,5 tetrazolium bromide)) assay. The A549, MCF-7, HeLa and HT-29 human cancer cells were seeded in 96-well plates at a density of 10^4^ cells/well (three replicates) and were left to adhere and grow overnight in a CO_2_ incubator (5%) at 37 °C. The 16HBE14o- and MCF10A cells were seeded at densities of 7.5 × 10^4^ and 4.5 × 10^4^ cells/well (three replicates), respectively, and left to adhere and grow overnight in the same conditions. The next day, the medium was collected and the cells were treated with the peptides (concentrations from 0 μM to 100 μM). Proliferation was determined after 48 h. Following the incubation period, 20 μL of MTT (5 mg/mL) was added to each well and incubated at 37 °C for 3.5 h. The reaction was stopped with the addition of 150 μL of a solution of 40 mM HCL in isopropanol. The MTT formazan formed was spectrophotometrically read at 590 nm in a microplate reader (SpectroStarNano, BMG LABTECH, Aylesbury, United Kingdom). Untreated cells were used as controls (0% of viability decrease) to determine the relative cell viability of treated cells.

### 2.5. LDH Release Assays

The Invitrogen^TM^ CyQUANT^TM^ LDH Cytotoxicity Assay Kit (Invitrogen, Waltham, MA, United States) was used to determine the LDH release of non-cancer cells treated with CT-p19LC, according to the manufacturer’s instructions. Briefly, 16HBE14o- and MCF10A cells were seeded at densities of 7.5 × 10^4^ and 4.5 × 10^4^ cells/well (three replicates), respectively, and left to adhere and grow overnight in the same conditions. The next day, the medium was collected and the cells were treated with the peptides (100 μM). After 48 h, the medium was collected and analyzed. Untreated cells were used as controls to compare the spontaneous LDH release and to normalize the data. Additional controls used were the maximum LDH activity release by lysing the cells with the lysis buffer provided in the kit, as well as the LDH positive control.

### 2.6. Quantitative Cellular Interaction

In order to evaluate the cell–peptide interaction, A549, MCF-7, HeLa and HT-29 cell lines were plated in 6-well plates with 5 × 10^5^ cells/well, respectively, and left to adhere and grow overnight in a CO_2_ incubator (5%) at 37 °C. The following day, the medium was removed, and the cells were washed twice with PBS and treated with 5 μM of CT-p19 and CT-p19LC labeled with 5,6-FAM over 2 h at 37 °C. After treatment, cells were washed twice with PBS, detached with TrypLE™ Express (Gibco^®^ by Life Technologies, Carlsbad, CA, United States) at 37 °C and resuspended in medium. Then, cells were collected by centrifugation at 1200 rpm over 3 min, washed once with PBS and re-dispersed in 350 μL of PBS for cytometry analysis.

The quantification of the peptides’ interaction with the cells was done using a BD Accuri™ C6 Plus Flow Cytometer (BD Biosciences, Devon, England), where peptides were detected through the fluorescein isothiocyanate (FITC) channel (FL1 detector, 533/563 nm; laser configuration of 3-blue 1-red, 640 nm laser). Measurements were carried out in triplicate and 20,000–50,000 events were acquired in the gated region of the forward-scatter/side-scatter plot per sample. A control based only on cells without treatment was also performed to exclude the possible cellular autofluorescence. The results were analyzed using the software FlowJo v10 by gating out cellular debris and doublets and expressed as the geometric mean fluorescence intensity (Geo MFI).

### 2.7. CT-p19LC Cellular Uptake

In order to characterize the cellular uptake of CT-p19LC, cells were cultured on µ-Slide 8-well glass-bottom chambers (ibidi^®^, Munich, Bavaria, Germany) with 5 × 10^4^ cells/well and left to adhere overnight before being treated with 5 µM of CT-p19LC-5,6-FAM peptide for 2 h. After this time, the medium was collected and the cells were washed twice with phosphate buffer saline (PBS) pH 7.4.Then, Alexa Fluor^®^ 633 WGA (Invitrogen™, Waltham, MA, United States; 1:200) and Hoechst 33342 (Invitrogen^TM^, Waltham, MA, United States; 1:500) were added to stain the plasma membrane and the nucleus, respectively, followed by 15 min of incubation. Finally, the samples were observed on a Leica TCS SP5 (Leica Microsystems CMS GmbH, Mannheim, Germany) inverted confocal microscope (model DMI6000) with a 63.3× water-immersion (1.2-numerical-aperture) apochromatic objective [34].

### 2.8. Apoptosis Assay

The FITC-Annexin V Apoptosis Detection Kit I (BD Pharmingen™, BD Biosciences, Devon, England) was used to study the apoptosis of cancer cell lines and non-cancer cell lines under study after treatment with CT-p19LC peptide. Briefly, A549, MCF-7, HeLa, HT-29 and the 16HBE14o- and MCF10A cell lines were plated in 6-well plates with 5 × 10^5^ and 7.5 × 10^5^ cells/well, respectively, and left to adhere and grow overnight in a CO_2_ incubator (5%) at 37 °C. The following day, the medium was removed and the cells were washed once with PBS pH 7.4 and treated with 20 μM of CT-p19LC over 48 h at 37 °C. After treatment, the cells were washed twice with PBS pH 7.4, detached with Accutase^®^ (Merck KGaA, Darmstadt, Germany) at 37 °C and resuspended in cell culture medium. After that, 1 × 10^5^ cell/mL was collected and centrifuged at 1200 rpm for 3 min. The supernatant was discarded, and cells were resuspended in 100 μL of 1X annexin V binding buffer. Then, FITC-annexin and PI (5 μL each) were added, and the cells were incubated at room temperature in the dark for 15 min. Finally, 400 μL of 1X annexin V binding buffer was added, and cells were analyzed on a BD Accuri™ C6 Plus Flow Cytometer (BD Biosciences, Devon, England). Untreated cells were used as a control. Cell death induction was considered by adding quadrant 2 (Q2) to quadrant 4 (Q4). At least 20,000 events were acquired and analyzed per sample.

### 2.9. Zeta Potential Measurements of Live A549, MCF-7, HeLa and HT-29 Cancer Cells and 16HBE14o- and MCF10A Non-Cancer Cells in the Presence of CT-p19LC

Zeta potential measurements through laser Doppler anemometry (LDA) were performed to assess the surface charge density of cancer and non-cancer cells and the electrostatic attraction of CT-p19LC toward them. For this, cells were diluted to 1 × 10^5^ cells/mL in DMEM and washed with PBS pH 7.4 twice (1200 rpm; 5 min). Then, cellular suspensions were incubated with different peptide concentrations (5, 10 and 20 μM) in serum-free medium for 30 min at 37 °C and dispensed into disposable zeta cells with gold electrodes. A set of 10 measurements (≈40 runs each) were performed at 37 °C with a voltage of 48 V (Malvern Instruments Ltd., Worcestershire, United Kingdom). Control values were obtained by measuring the surface charge of each cellular suspension in the absence of CT-p19LC (0 μM, untreated condition).

### 2.10. GP Determination for Membrane Order Evaluation

The membrane order evaluation of the A549, MCF-7, HeLa and HT-29 human cancer cell lines after CT-p19LC treatment was investigated with the probe Laurdan using two-photon excitation microscopy. Cells were treated for 2 h with 20 µM of CT-p19LC after seeding with 5 × 10^4^ cells on µ-Slide 8-well glass-bottom chambers (ibidi^®^, Munich, Bavaria, Germany). Subsequently, two washing steps with PBS pH 7.4 were performed followed by incubation at 37 °C for 15 min with medium containing 5 μM of Laurdan [35]. Untreated cells were used as controls. Following incubation, samples were examined on a Leica TCS SP5 inverted confocal microscope (model DMI6000) with a 63.3× water-immersion (1.2-numerical-aperture) apochromatic objective. Fluorescence microscopy data was obtained by using a titanium-sapphire laser as the excitation light source (the wavelength was set to 780 nm and the fluorescence emission was collected at 400–460 nm and 470–550 nm to calculate the GP images). Fluorescence imaging data was processed through homemade software based on a MATLAB environment, with the GP value defined as GP = (/400–460−G.I470-530)/(/400–460 + G.I470-530). The parameter G was a calibration factor calculated from imaging Laurdan in DMSO (GP = 0.01 in this solvent) using the same experimental conditions.

### 2.11. Statistical Analysis

Statistical analysis was performed using GraphPad Prism 8.0.1 (GraphPad Software Inc., San Diego, CA, United States). Statistical significance of the difference between two groups was evaluated by with Student’s *t*-test. Differences between groups were compared using one-way analysis of variance (ANOVA) and Tukey’s multiple comparisons test. Results are expressed as means ± standard deviation (SD) and geometric means with 95% confidence intervals.

## 3. Results and Discussion

### 3.1. CT-p26 Peptide Effect on Cell Viability Confirms the Anticancer Potential of C-Terminal Azurin

The CT-p26 peptide comprises amino acid residues 95 to 120, close to the C-terminal region of the bacterial protein azurin (Table 1), which is known to contribute to its anticancer activity as well as to its ability to enter cancer cells [28,29,30].

**Figure 1:**
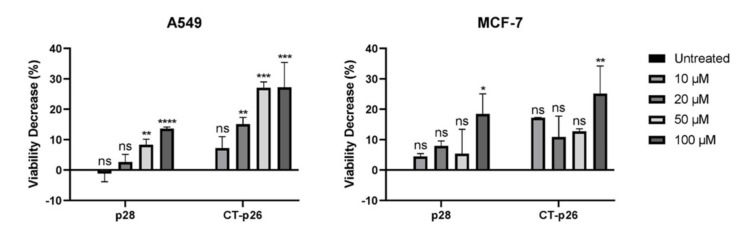
Comparison of cell viability after treatment with p28 and CT-p26 peptides (0 to 100 μM) in A549 (lung) and MCF-7 (breast) cancer cells incubated over 48 h. Untreated condition (control) consisted of cells incubated with medium only. Values represent the means ± SD, and each condition had at least n = 3. *, **, ***, **** and ns denote significant differences of *p* < 0.1, *p* < 0.01, *p* < 0.001 and *p* < 0.0001 and differences that were not statistically significant, respectively, when comparing control with treatments.

Taking this into account, MTT cell proliferation assays were performed to evaluate the effect of this peptide on A549 lung and MCF-7 breast cancer cell lines. Parallel assays, under the same conditions, have also been carried out with the p28 peptide, also derived from azurin and mentioned previously for its anticancer properties [17,21,25]. These assays were performed with increasing concentrations of both peptides, from 0 to 100 µM. As shown in Figure 1, the two peptides exhibited cytotoxic activity against both cancer cell lines, and a dose–response effect is evident in the A549 lung cancer cell line. Moreover, treatment with CT-p26 leads to a higher decrease in cell viability than treatment with p28: by about two- to seven-fold in the case of A549 cells and one- to four-fold in the case of MCF-7 cells. These results confirmed that the C-terminal region of the azurin protein can be used as an anticancer functional peptide, thereby making it an interesting lead peptide.

### 3.2. CT-p19 Peptide Decreases Cancer Cell Viability and Has Selective Property

**Figure 2:**
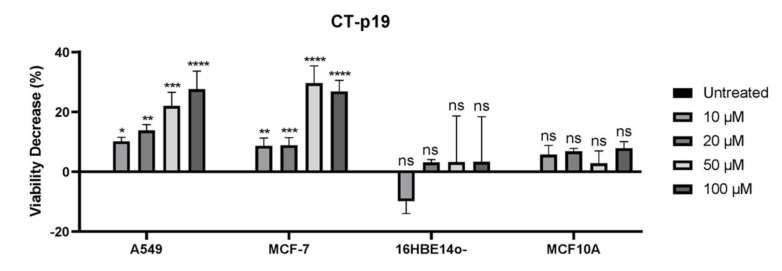
Viability decrease (100% of proliferation in untreated condition—% of proliferation for each treatment condition) of A549 (lung) and MCF-7 (breast) cancer cells and 16HBE14o- (bronchial) and MCF10A (mammary gland) non-cancer cells when incubated with different concentrations of CT-p19 peptide (0 to 100 μM) over 48 h. Untreated condition (control) consisted of cells incubated with medium only. Values represent the means ± SD, and each condition had at least n = 3. *, **, ***, **** and ns denote significant differences of *p* < 0.1, *p* < 0.01, *p* < 0.001 and *p* < 0.0001 and differences that were not statistically significant, respectively, when comparing control with treatments.

The in silico study previously performed by our group made it possible to design a new peptide with a shorter length, and with a higher propensity to demonstrate anticancer activity, from the C-terminal peptide (support vector machine (SVM) score: 0.76 vs. 0.90; Table 1) [31]. This parameter and the possible selectivity of this peptide, as seen in azurin and the other peptides derived therefrom [25,36], were evaluated through an MTT cell proliferation assay on the cancer cell lines under study and on two matching-tissue non-cancer cell lines, 16HBE14o- and MCF10A (Figure 2). After treatment with 10, 20, 50 and 100 μM of CT-p19, decreases in cell viability of 10%, 14%, 22% and 28% were observed in the case of the A549 cancer cell line. The same concentrations of CT-p19 induced decreases of 9%, 9%, 30% and 27% on the viability of MCF-7 cells. Regarding non-cancer cell lines, the viability decrease did not exceed 3% in 16HBE14o- and 8% in MCF10A. Thus, the results showed that the CT-p19 peptide is able to decrease the viability of cancer cells but not of non-cancer cells, which demonstrates that this peptide has the desired selectivity. These results provided a smaller version of the CT-p26 peptide while maintaining its anticancer activity.

### 3.3. The Newly Designed CT-p19LC Peptide Reduces Proliferation and Induces Cell Death in Cancer Cell Lines

After the development of CT-p19 in silico, our group re-designed a new peptide based on single substitutions of amino acid residues that made it possible to not only increase the SMV score to 0.99 but also improve its solubility (Table 1). Thus, this new peptide, designated CT-p19LC, contained 19 amino acids (VSKLRKGEKYMFFCTFPGH) and represented an iterative peptide optimization from a region close to the C-terminal of the anticancer protein azurin. It had a molecular weight of 2275.7 g/mol (2.3 kDa), a pI of pH 10 and a net charge of +3.5 at pH 7 (Table 1) [31].

**Figure 3:**
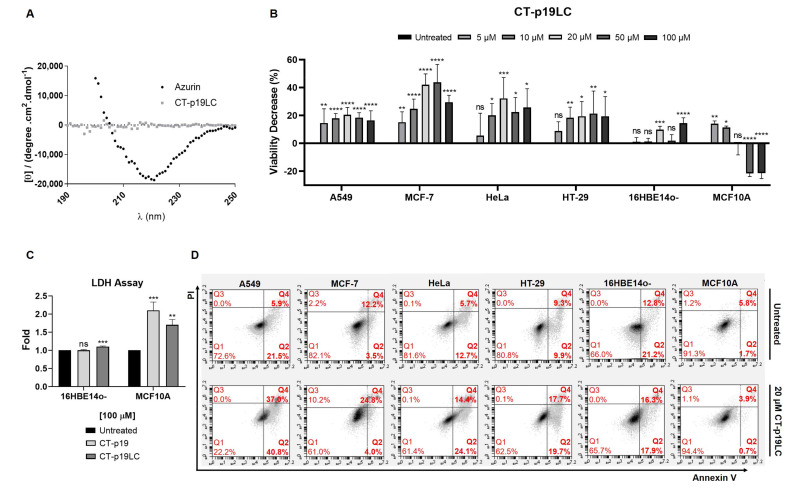
Cytotoxic effect of newly designed CT-p19LC peptide: (**A**) circular dichroism spectra of azurin and CT-p19LC (5 µM) in sodium phosphate buffer 10 mM, pH 7.4, at 25 °C; (**B**) viability decrease (100% of proliferation in untreated condition—% of proliferation for each treatment condition) of A549 (lung), MCF-7 (breast), HeLa (cervix) and HT-29 (colorectal) cancer cells and 16HBE14o- (bronchial) and MCF10A (mammary gland) non-cancer cells when incubated with different concentrations of CT-p19LC peptide (0 to 100 μM) over 48 h. Untreated condition (control) consisted of cells incubated with medium only. Values represent the means ± SD, and each condition had at least n = 3. *, **, ***, **** and ns denote significant differences of *p* < 0.1, *p* < 0.01, *p* < 0.001 and *p* < 0.0001 and differences that were not statistically significant, respectively, when comparing control with treatments; (**C**) LDH assay in non-cancer cell lines treated with 100 μM of CT-p19 and CT-p19LC. Values represent the means ± SD (n = 3). **, *** and ns denote significant differences of *p* < 0.01 and *p* < 0.001 and differences that were not statistically significant, respectively, when comparing treatments with control; (**D**) apoptosis assay in cancer and non-cancer cells treated with 20 μM of CT-p19LC for 48 h, assessed by flow cytometry. Representative figures showing a population of viable cells in the left lower quadrant (Q1; annexin V − /PI −), early apoptotic cells in the right lower quadrant (Q2; annexin V+/PI −), necrotic cells in the left upper quadrant (Q3; annexin V−/PI+) and advanced apoptotic or necrotic cells in the right upper quadrant (Q4; annexin V+/PI+).

In this work, circular dichroism (CD) spectral measurements (Figure 3A) indicated that the peptide adopted a randomly coiled structure in solution.

To evaluate the anticancer potential of the CT-p19LC peptide, MTT cell proliferation and apoptosis assays were carried out. For this, the spectrum of cell lines used was expanded by adding the HeLa (cervix) and HT-29 (colorectal) cancer cell lines to the A549 (lung) and MCF-7 (breast) cancer cells, and the 16HBE14o- (bronchial) and MCF10A (mammary gland) non-cancer cell lines.

First, the MTT cell proliferation assays were performed with increasing doses of CT-p19LC (0 to 100 µM; Figure 3B). Comparing it with the CT-p19 treatment that led to a dose–response effect on the lung and breast cancer cell lines (Figure 2), this same effect was only observed at the lowest concentrations of 5, 10 and 20 µM in the case of the CT-p19LC treatment. At higher concentrations of 50 and 100 µM, a stabilization of the decrease in viability was observed. However, we observed that for the concentration of 20 μM of CT-p19LC, the values for the decrease in viability were similar to those obtained with higher concentrations of CT-p19. These results confirmed the anticancer potential predicted in silico for CT-p19LC (0.90 vs. 0.99 SMV score; Table 1). The CT-p19LC treatment in the cervix and colorectal cancer cell lines demonstrated that this peptide can exert its anticancer action on a wide spectrum of cancer lines, since a decrease in cell viability of 20–30% was observed (Figure 3B). It is interesting to note that the values of the decrease in viability for the concentration of 20 μM of CT-p19LC in all cancer cell lines were around 20–40%, and to achieve the same decrease with the azurin (Table 1) or p28 peptide treatment (Figure 1), 100 μM would be needed. Furthermore, it was also found that CT-p19LC does not have a cytotoxic effect on the non-cancer cell lines under study (in all concentrations tested, less than a 14% decrease in viability was observed; Figure 3B), which indicates that this peptide also demonstrates selectivity for cancer cells, an important and desired characteristic in the development of new anticancer compounds. The non-toxic effect on non-cancer cells was also supported by the low levels of spontaneous LDH release in cells treated with 100 µM of peptide, in particular for CT-p19LC (Figure 3C).

Second, the apoptosis assays supported the MTT cell proliferation assays. Treating cancer cells with a single dose of CT-p19LC at 20 μM strongly promoted cell death. This concentration was chosen as it corresponded to the maximum anticancer potential, since higher concentrations had no additional impact on cell viability. In A549 cells, there was induction of cell death in 77.8% of the cells, in MCF-7 in 28.8%, in HeLa in 38.5% and in HT-29 in 37.4%, which were comparable to the values in their controls (untreated condition) of 27.4%, 15.7%, 18.4% and 19.2%, respectively (Figure 3D). Importantly, the same was not observed in non-cancer cell lines, since in 16HBE14o- (34.0% control condition vs. 34.2% treatment condition) and MCF10A (7.5% control condition vs. 4.6% treatment condition), cell death was similar to the control condition (untreated), again demonstrating the selectivity of this peptide (Figure 3D). Overall, these results indicate that CT-p19LC induces a decrease in cell viability in part through the induction of cell death.

### 3.4. CT-p19LC Peptide Targets Cellular Plasma Membrane

**Figure 4:**
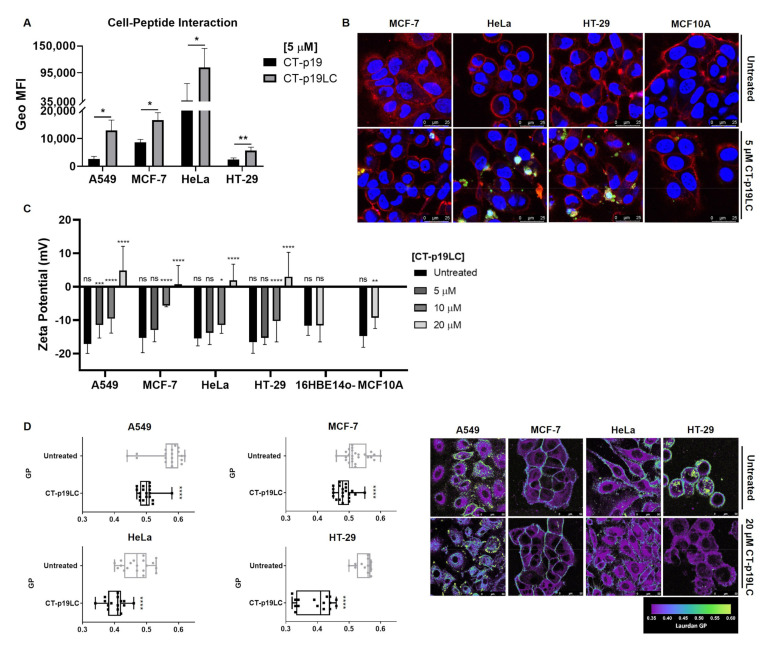
CT-p19LC membrane-active properties: (**A**) flow cytometry quantitative analysis of cancer cell–peptides interaction. Results are reported as the means ± SD, and each condition had at least n = 3. * and ** denote significant differences of *p* < 0.1 and *p* < 0.01, respectively, when comparing CT-p19LC treatment with CT-p19 treatment; (**B**) representative confocal microscopy qualitative analysis of CT-p19LC cellular uptake by MCF-7, HeLa and HT-29 cancer cells and MCF10A non-cancer cells incubated with PBS pH 7.4 as control and 5 μM of peptide labeled with 5,6-FAM (green color) for 2 h. WGA Alexa Fluor^®^ 633 and Hoechst 33342, for staining the plasma membrane and nucleus, respectively, are shown in red and blue colors. Scale bars represent 25 μm; (**C**) zeta potential of A549, MCF-7, HeLa and HT-29 cancer cells and 16HBE14o- and MCF10A non-cancer cells in the presence of CT-p19LC peptide. A total of 1.5 × 10^5^ cells/mL were incubated and stabilized for 30 min at 37 °C with different peptide concentrations, and the zeta potential was measured. Data are represented as means ± SD. *, **, ***, **** and ns denote significant differences of *p* < 0.1, *p* < 0.01, *p* < 0.001 and *p* < 0.0001 and differences that were not statistically significant, respectively, when comparing the untreated condition (0 μM) with increasing concentrations of CT-p19LC (5, 10 and 20 μM); (**D**) the effects of CT-p19LC on the cell‘s membrane order for A549, MCF-7, HT-29 and HeLa cancer cell lines and their respective GP values. All represented cell lines were seeded on μ-Slide 8-well glass-bottom chambers and treated with 20 μM of CT-p19LC for 2 h. For each condition, 5 μM of Laurdan was used. Untreated cells were used as the control. Homemade software built in a MATLAB environment was used to measure the GP values. Representative Laurdan GP images are shown. Scale bars represent 50 μm. Average GP values are expressed as means ± SD from at least 15 individual cells in each condition. Results are compared to the untreated population with equal variance (****, *p* < 0.0001).

It is known that the plasma membrane of cancer cells is more anionic at their surface than for non-cancer cells due to its constitution based on negatively charged components [3,4,5]. In addition, one of the mechanisms by which it has been proposed that there is an electrostatic attraction of ACPs towards this cellular barrier of cancer cells is related to the positive charge of these peptides [4,10]. In the case of the CT-p19LC peptide, the in silico approach established a charge of +3.5 at pH 7 (Table 1) [31]. Therefore, we evaluated the capacity of CT-p19 and CT-p19LC to associate to the cancer cell lines using flow cytometry. Cells were treated with 5,6-FAM labeled peptides (5 µM) and left to interact with the cells for 2 h. A stronger association of CT-p19LC was observed for all cell lines compared to CT-p19, which may have contributed to its higher anticancer activity (Figure 4A). We then proceeded to analyze the cellular distribution of this peptide in both cancer and non-cancer cells using fluorescence confocal microscopy (Figure 4B). The peptide was detected both in the plasma membrane and intracellularly distributed, suggesting its capacity to penetrate the plasma membrane and even reach the nucleus, but only in cancer cells. In the non-cancer cell line MCF10A, almost no peptide was detected.

We also evaluated the zeta potential of the live non-cancer and cancer cell lines under study in the presence of increasing concentrations of CT-p19LC peptide (0 to 20 μM). The measurements of the zeta potential allowed the assessment of the electrostatic potential that is triggered after a particle with a certain charge is placed in solution with others [37]. This concept can be applied to evaluate the interaction of peptides with cell membranes, which results in the alteration of the cell surface electropotential [38]. As the concentration of CT-p19LC exposed to cancer cells increased, an increase in the zeta potential was obtained in all cancer cell lines, with this potential reaching positive values for the highest concentration of the peptide (Figure 4C).

After treatment with 20 µM of CT-p19LC, the potential of the lung cancer cell line increased from −17.2 ± 2.8 mV to 4.8 ± 7.3 mV; in the case of the breast cancer cell line, it increased from −15.4 ± 4.4 mV to 0.8 ± 5.5 mV; in the cervix cancer cell line, it increased from −15.5 ± 2.2 mV to 1.9 ± 4.8 mV; and, finally, in the colorectal cancer cell line, it increased from −16.6 ± 3.3 mV to 3.0 ± 7.3 mV. These results indicate that this peptide targets the plasma membrane of cancer cells. In the case of the non-cancer cell lines, at the highest concentration used (20 µM), it was found that the potential remained negative and close to the value obtained in the untreated condition (Figure 4C). For 16HBE14o-, before treatment the zeta potential was −11.7 ± 2.9 mV, and it did not change with the treatment (−11.6 ± 4.9 mV). In the case of MCF10A, before treatment the zeta potential was −14.8 ± 3.3 mV, and after treatment it increased only moderately to −9.3 ± 3.2 mV, remaining more negative than that obtained in the same concentration of peptide in cancer cells. Thus, these results show that the CT-p19LC peptide directs itself towards the cancer cell membranes much more strongly than towards non-cancer cell membranes.

To further characterize the effect on the membranes of cancer cells, the membrane order of the plasma membranes subjected to treatment with the CT-p19LC peptide was investigated with the Laurdan probe using two-photon excitation microscopy. To do this, the cancer cells (A549, MCF-7, HT-29 and HeLa) were treated over 2 h with CT-p19LC at 20 µM. To quantify the degree of lipid packing (the measured mean of the GP value) in both conditions (untreated and treated cancer cells), homemade software created in a MATLAB environment was used. The GP value varies between −1 and 1; a GP value higher than 0.5 indicates the existence of very compact and ordered membranes. In contrast, a GP value lower than 0.5 is typically observed for more fluid membranes [35,39]. As shown in Figure 4D, for the four cancer cell lines the GP values decreased after CT-p19LC treatment, making the cell membranes more fluid (A549: 0.57 to 0.50; MCF-7: 0.53 to 0.48; HeLa: 0.47 to 0.40; HT-29: 0.55 to 0.39). This common pattern indicates that the CT-p19LC peptide acts efficiently at the plasma-membrane level. Fluorescence microscopy images of the cells showed that treated cells suffered a variety of morphological modifications; i.e., the cell shape became irregular and the fragmentation of the plasmatic membrane and the nucleus was visible (Figure 4D).

In general, these results indicate that the CT-p19LC peptide engaged with the plasma membrane, which could trigger the apoptotic events. However, it remains to clarify the possible membrane components that could be targets of CT-p19LC. Further studies with biophysical approaches such as atomic force microscopy (AFM) or leakage studies using model membranes (liposomes) are necessary to unravel the mode of action of this peptide against cancer cells.

## 4. Conclusions

The CT-p19LC anticancer potential explored in this work reinforces the relevance of studies in other domains of azurin that contain anticancer properties of their own. In an initial approach, a region of the C-terminal domain of azurin, which was studied in the form of a peptide with 26 residues, CT-p26, was shown to have a similar anticancer potential to the p28 peptide and azurin. From here, the in silico redesign of this region made it possible to decrease the length of its peptide chain and increase its anticancer potential, as well as its selectivity for cancer cells through changes in hydrophobicity and net charge, giving rise to a new peptide called CT-p19LC. The results of this work suggest that the CT-p19LC application induced a decrease in the cell viability, in part through the triggering of cell death, in all the cancer cell lines under study, without affecting the non-cancer cell lines. In addition to this, it was also demonstrated that this peptide selectively binds to the plasma membranes of cancer cells, since its electrostatic potential is altered, and changes occur at the level of lipid packing. All in all, this study characterizes CT-p19LC as a synthetic ACP with improved and selective anticancer potential and with membrane-active properties.

## Figures and Tables

**Table 1:** Overview of the characteristics of azurin and its derived peptides.

PROTEIN/ PEPTIDE	STRUCTURE	AMINO ACID SEQUENCE	AZURIN POSITION	HYDROPHOBICITY	CHARGE	ISOELECTRIC POINT	WATER SOLUBILITY	SMV SCORE	VIABILITY DECREASE AFTER 100 μM OF PROTEIN/ PEPTIDE TREATMENT	REFERENCES
**Azurin**	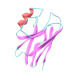	128 aa	n.a.	n.a.	n.a.	n.a.	n.a.	n.a.	20–40%	[30]
**p28**	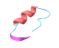	LSTAADMQGVVTDGMASGLDKDYLKPDD	50–77 aa	n.a.	n.a.	n.a.	n.a.	n.a.	0–25%	[21]
**CT-p26**	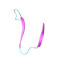	VTFDVSKLKEGEQYMFFCTFPGHSAL	95–120 aa	−0.03	−0.5	5.3	Poor	0.76	n.a.	[31]
**CT-p19**	n.a.	VSKLKEGEQYMFFCTFPGH	99–117 aa	−0.08	0.5	7.0	Poor	0.90	n.a.	[31]
**CT-p19LC**	n.a.	VSKLRKGEKYMFFCTFPGH	n.a.	−0.16	3.5	10.0	Good	0.99	n.a.	[31]

SMV: Support vector machine score; aa: amino acids; n.a.: not applicable

## Data Availability

The data presented in this study are available on request from the corresponding author.
